# High-Performance Polyacrylic Acid-Grafted PVDF Nanofiltration Membrane with Good Antifouling Property for the Textile Industry

**DOI:** 10.3390/polym12112443

**Published:** 2020-10-22

**Authors:** Yu-Hsuan Chiao, Shu-Ting Chen, Micah Belle Marie Yap Ang, Tanmoy Patra, David Alfonso Castilla-Casadiego, Rong Fan, Jorge Almodovar, Wei-Song Hung, S. Ranil Wickramasinghe

**Affiliations:** 1 Ralph E. Martin Department of Chemical Engineering, University of Arkansas, Fayetteville, AR 72701, USA; ychiao@uark.edu (Y.-H.C.); sc068@uark.edu (S.-T.C.); tpatra@uark.edu (T.P.); dac011@uark.edu (D.A.C.-C.); jlalmodo@uark.edu (J.A.); 2 Graduate Institute of Applied Science and Technology, National Taiwan University of Science and Technology, Taipei 10607, Taiwan; 3 R&D Center for Membrane Technology and Department of Chemical Engineering, Chung Yuan University, Chung Li 32023, Taiwan; mbmyang@gmail.com; 4 Institute of Bioprocess Engineering and Pharmaceutical Technology, University of Applied Sciences Mittelhessen, 35390 Giessen, Germany

**Keywords:** poly-(acrylic acid), loose nanofiltration, UV-grating, dye removal, superoleophobic

## Abstract

In the textile industry, a high-efficiency dye removal and low-retention of salt is demanded for recycling wastewater. In this study, polyvinylidene fluoride (PVDF) ultrafiltration membrane was transformed to a negatively charged loose nanofiltration (NF) membrane through UV-grafting of acrylic acid. At the optimal exposure of PVDF membrane in UV light for 5 min, the membrane had a high dye recovery above 99% (Congo red and Eriochrome^®^ Black T) and a low sodium chloride (NaCl) rejection of less than 15% along with pure water flux of 26 L∙m^−2^∙h^−1^∙bar^−1^. Its antifouling and oleophobicity surface properties were verified using fluorescent- bovine serum albumin (BSA) and underwater mineral oil contact angle, respectively. According to the fluorescent microscopic images, the modified membrane had ten times lower adhesion of protein on the surface than the unmodified membrane. The underwater oil contact angle was raised from 110° to 155°. Moreover, the salt rejection followed this sequence: Na_2_SO_4_ > MgSO_4_ > NaCl > MgCl_2_, which agreed with the typical negatively charged NF membrane. In addition, the physicochemical characterization of membranes was further investigated to understand and link to the membrane performance, such as surface functional group, surface elements analysis, surface roughness/morphology, and surface hydrophilicity.

## 1. Introduction

In recent years, dye removal from textile wastewater is considered as a critical issue because of the potential environmental hazard and associated difficulties in terms of dye recycling [1]. Discharging of dye into the environment even at low concentration will endanger aquatic life with a significant reduction in their photosynthetic activity due to the enhanced toxicity in water [2]. Various technologies have been studied for efficient degradation or removal of dyes from the aqueous phase; however, most of them are neither cost-efficient nor scalable for industrial applications [3,4,5]. Other treatment methods, such as biological, chemical treatment, and catalytic degradation, were also investigated for the dye removal from wastewater [6,7]; however, the expensive cost and less scale-up possibility are the two factors limiting their application in terms of commercialization. In this context, membrane technology is considered as a promising solution for textile wastewater due to high-quality production without sludge generation and relatively low operational cost compared to the nonphysical separation process.

Loose nanofiltration (NF) is a pressure-driven membrane separation technique that can be used in various applications such as water and wastewater treatment, antibiotic separation, biopurification, and biomass recovery [8,9,10,11,12,13,14]. Its separation capability is in between reverse osmosis and ultrafiltration, which are used to remove or concentrate the multivalent and divalent salts or small molecular components. Moreover, the requirement of hydraulic pressure is lower than reverse osmosis, which demands a hydraulic pressure to overcome the osmotic pressure of the feed stream. The separation mechanism of the NF membrane comprises synergistic sieving effect and charge effect (Donnan exclusion) [13,15,16]. Thus, during optimal design of such NF membrane-based processes, one must consider the specific separation mechanism between the membrane and components in the feed.

The occurrence of membrane fouling still remains as a major problem in industrial separation processes because it leads to reduction of the membrane efficiency. Typically, in water treatment, the membrane fouling is classified into particulates: organic, inorganic, and biofouling [17]. Herein, understanding the mechanism of membrane fouling for a particular application is important in order to improve the fouling resistance of the membrane. Yu [18] has already investigated the extracellular polymeric substances (EPS)-pollutant interaction and suggested that much attention must be paid to the EPS and living cells as key parameters in wastewater treatment. Transparent exopolymer particles are commonly present in the water source, which can easily form a gel layer on the membrane surface and confound each particle between the cations [19]. However, based on the understanding of the fouling mechanism, fabrication of antifouling membrane was also required simultaneously to be explored and addressed for future applications.

To date, several modifications have been proposed to prepare highly selective NF membranes with good antifouling property, such as interfacial polymerization, self-assembly of polyelectrolyte, polymer coating, and UV-grafting [6]. Interfacial polymerization (IP) is the most common technique employed for producing a commercial thin-film composite (TFC) membrane [8,20]. Within a short reaction time, a thin and dense selective layer can be acquired. For self-assembly of polyelectrolyte, the thickness of the active layer is easily controlled, and stacked by the electrostatic interaction [21]. Huang et al. report deposition of the 2-hydroxypropyltrimethyl ammonium chloride chitosan (HACC) into the polyacrylonitrile (PAN) membrane. Their modified membrane is immersed into the toluene diisocyanate (TDI) solution for further crosslinking to create a dense selectivity layer [22].

Apart from these methods, UV-grafting is another method to modify the surface of the membranes. It has been widely used for fabricating a stable selective layer [23,24]. The graft polymerization through UV is fast and inexpensive. Furthermore, a less toxic solvent is used in this method [25,26]. Earlier studies using UV-grafting report several applications with enhanced surface antifouling and hydrophilic properties by tuning the surface charges [24,27,28,29]. Zhong et al. [23] report a positively charged NF membrane fabricated with [2-(methacryloyloxy)ethyl]trimethyl ammoniumchloride and diallyldimethyl ammonium chloride on the sulfonated polyphenylenesulfone (sPPSU) through UV-grafting method. Their salt rejection sequence follows a typical positively charged membrane, and up to 99.98% recovery of the Safranin O dye.

In this study, a novel loose NF membrane was prepared from the commercial PVDF ultrafiltration membrane using UV-grafting technique for dye removal. Herein, the prepared NF membrane with low salt rejection was designed using functionalized surface entities for efficient recycling and recovery of the dye without NaCl. Moreover, acrylic acid (AA) was used to generate the selectivity layer, with benzophenone (BP) as a photoinitiator. The improvement of selectivity between dye and salt was investigated with systematic optimization of the UV exposure time. Furthermore, the antifouling property was tested using the fluorescent-bovine serum albumin (BSA) protein attachment test and the lysozyme adsorption test. To further understand the mechanism of membrane separation after modifying the membrane, the superoleophobicity of the surface and its physicochemical characterization were also analyzed. Additionally, the Donnan exclusion phenomenon was simultaneously explored to optimize the salt rejection in the presence of the negatively charged O-atom of carboxylic acid group on the AA.

## 2. Material and Methods

### 2.1. Materials

Methanol, ethanol, benzophenone (BP), MgSO_4_, NaCl, MgCl_2_, NaSO_4,_ acrylic acid (AA), and phosphate-buffered saline (PBS) tablets were purchased from VWR (Atlanta, GA, USA). Bovine serum albumin (BSA) was supplied by Lee BioSolution (Maryland Heights, MO, USA). Congo red and Eriochrome^®^ Black T(EBT) were procured from Sigma-Aldrich (St. Louis, MO, USA). DI water was used in all experiments. Commercial polyvinylidene fluoride (PVDF) 400kDa membrane was provided by the UltraTM, Oceanside, CA. Commercial nanofiltration membrane NF270, NF90, and NF245 were provided by DuPont Water Solutions (Midland, MI, USA).

### 2.2. NF Membrane Fabrication

The commercial membrane was soaked in 50% ethanol/DI water for at least 3 h to remove the impurities and glycerin on the membrane surface. The wet membrane sample was rinsed with DI water 3 times, followed by drying in a vacuum oven at 30 °C for at least 12 h. Similar to our previous work, the surface of PVDF ultrafiltration membrane was modified through UV-grafting technique [27,30]. Briefly, the membrane was immersed in 2% BP/methanol solution for 30 min, which was used as the photoinitiator to generate the free-radical on the membrane. Afterward, 5 *w*/*v*% of AA solution was poured into the Petri dish. Furthermore, the membrane and AA solution were covered using a clean quartz plate and placed in the UV reactor for a certain exposure time. The 365 nm wavelength UV lamp (UVaprint 100, Honle UV American Inc, Marlborough, MA, USA) intensity had ~43 mW/cm^2^, and the distance between the lamp and the sample was fixed at 30 cm. The resulting membranes were thereafter rinsed at least 3 times using methanol and DI water to eliminate residual chemicals; furthermore, the membrane was washed using DI water for at least 24 h before any subsequent testing or characterization to ensure no weakly bound AA was present on the membrane.

### 2.3. Characterization of NF Membranes

The resulting membranes were dried in a vacuum oven overnight before characterization. Attenuated total reflectance–Fourier transform infrared spectroscopy (ATR–FTIR, Perkin Elmer Spectrum 100 FT–IR Spectrometer, Waltham, MA, USA), and X-ray photoelectron spectrometry (XPS, Thermo Fisher Scientific Inc., Waltham, MA, USA) were used to analyze the functional groups and chemical composition of the membrane surface. The hydrophilicity and oleophobic properties were measured by a contact angle measurement instrument (model OCA15EC, Future Digital Scientific, Garden City, NY, USA). The surface roughness and morphology were observed using an atomic force microscope (AFM) purchased from Bruker, CA, USA. The scanning electron microscope (SEM, FESEM S-4800) applied in this investigation was obtained from Hitachi Co., Tokyo, Japan.

### 2.4. Performance of the NF Membranes

NF membrane performance was tested using a custom-made crossflow system. The effective membrane area in the circular stainless-steel module was ~12 cm^2^ without the spacer [8]. The membrane was compacted at 5 bar for 1 h prior to subsequent operation at 4 bar. The temperature and flow rate were controlled at ~23 °C and 0.6 L/min, respectively. The water flux, *J* was calculated using Equation (1).

(1) $$Flux\;\left(J\right)=\frac{g}{\rho \times A\times time\times bar}$$

where *g* is the mass of permeate recorded at a certain period of time, A is the effective membrane area in the cell, and ρ is the density of water.

The salt rejection (1000 mg/L of Na_2_SO_4_, MgSO_4_, MgCl_2_, NaCl solution) and dye removal (100 mg/L of Congo red and EBT) were monitored using a conductivity meter (WTW, Weilheim, Germany) and a UV-Vis spectroscope (Thermo Fisher Scientific, Waltham, MA), respectively. Equation (2) was used to obtain the rejection of the solute. The detected wavenumber of Congo and EBT are 490 and 503 nm.

(2) $$Rejection,\;R\;\%=\left(1-\frac{C_{p}}{C_{f}}\right)\times 100\%$$

where $C_{p}$ and $C_{f}$ are the concentration of the feed and permeate in the tank, respectively.

### 2.5. Fluorescent BSA Attachment and Lysozyme Adsorption Test

The membrane antifouling behavior was visualized using fluorescent conjugated BSA. In a typical experiment, 0.1 g/L florescent BSA was prepared in a prefilter PBS buffer and gently shaken to avoid protein deformation. The membrane sample was cut and placed into a sample jar. The jar was then shaken in the incubator for 4 h at 60 rpm. Afterward, the membrane was rinsed three times using PBS buffer, carefully put on a microslide, and covered by a smaller microslide sealing using nail polish. The image was taken using an inverted laboratory microscope, Leica DM IL LED purchased from Leica (Wetzlar, Germany). The surface BSA coverage statistical quantification analysis result was obtained using ImageJ^®^ software [31].

For the lysozyme static adsorption test, the membrane was cut using a 2.54 cm diameter circular stainless-steel punch. The membrane was washed with PBS buffer solution for at least one day to swell the membrane and to remove any impurities. Afterward, the membrane was dipped into a 0.1 mg/mL lysozyme PBS buffer solution in an incubator for 5 h. The content of protein before and after the adsorption test was analyzed using a UV-Vis spectroscopy (GENESYS™ Vis/UV-Vis Spectrophotometers, Thermo Fisher Scientific, Waltham, MA) at absorption wavenumber 280 nm [32]. The membrane lysozyme adsorption ability was calculated as follows:

(3) $$\mathrm{Adsorption}\;\mathrm{ability}=\frac{\mathrm{Initial}\;\mathrm{protein}\;\mathrm{concentration}-\mathrm{protein}\;\mathrm{concentration}\;\mathrm{after}\;\mathrm{membrane}\;\mathrm{adsorption}}{\mathrm{membrane}\;\mathrm{surface}\;\mathrm{area}}$$

## 3. Results and Discussion

### 3.1. Physicochemical Properties Characterization of the Loose NF Membranes

**Figure 1:**
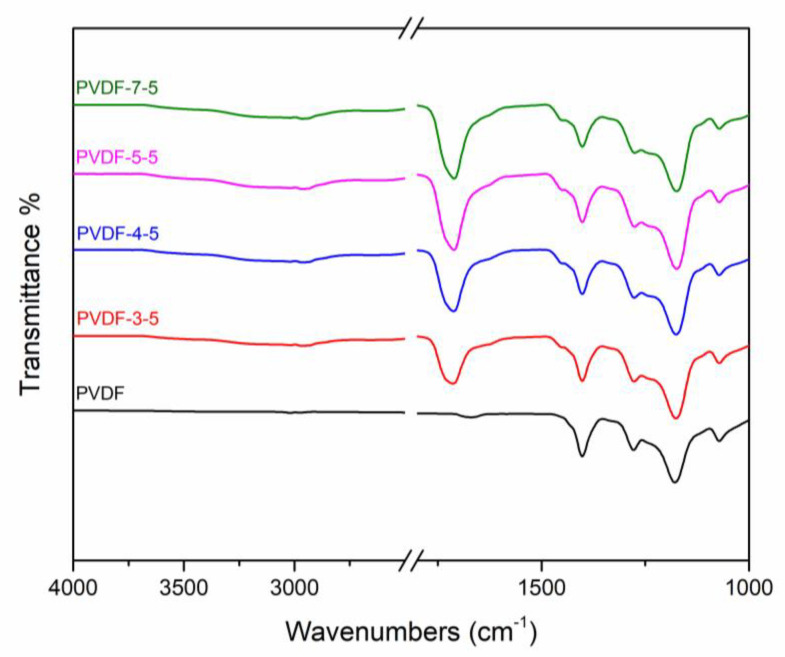
ATR–FTIR spectrum of unmodified membrane (PVDF) and modified membranes (PVDF-3-5, PVDF-4-5, PVDF-5-5, PVDF-7-5). The acrylic acid (AA) monomer concentration was 5 *w*/*v*%, and the benzophenone (BP) photoinitiator concentration was kept at 2 *w*/*v*%.

In the scope of this study, a loose NF membrane was fabricated using the UV-grafting surface modification method. The loose nanofiltration membrane was developed and reported in an earlier study [33], where the membrane consisted of a hydrophilic surface with loose selectivity layer structure and small water channels [34]. The selectivity layer was generated using polymerization of AA on the PVDF support membrane. Several characterization methods were employed to verify the membrane physicochemical properties. Figure 1 depicts the ATR–FTIR spectrum of the resulting membrane. The clear vibration peaks of PVDF at 1275 and 1170 cm⁻^1^ are attributed to the symmetric and asymmetric stretching of CF_2_, respectively [27,35]. Due to the vibration of CH_2_ on the PVDF polymer, the significant peak at 1400 cm⁻^1^ is also evident in the spectrum [36]. As compared to the unmodified PVDF, the new peak at 1710 cm⁻^1^ on the modified membrane signifies the successful grafting of carboxylic acid group (C=O) of the poly-AA (PAA) layer [37]. Overall, the ATR–FTIR result confirms that the PAA was successfully generated on the support. XPS elemental analysis is listed in Table 1, which would additionally verify that the PAA fully covered the PVDF surface. Absence of the signal for elemental F in case of the modified membranes and the presence of elemental O corresponding to the carboxylic acid of AA confirm the successful surface modification.

**Figure 2:**
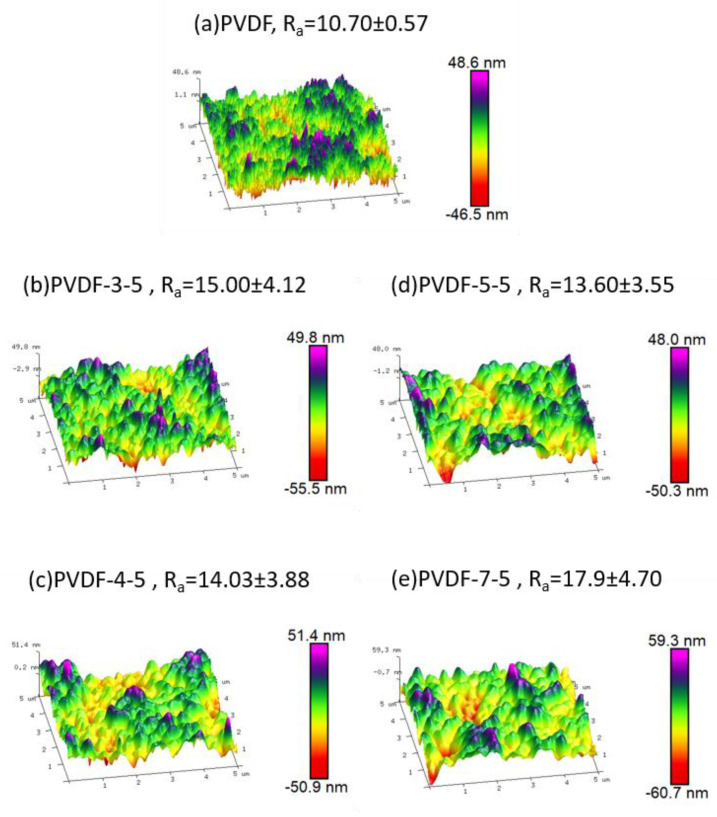
Three-dimensional (3D) AFM images of the loose NF membrane. (**a**) PVDF, (**b**) PVDF-3-5, (**c**) PVDF-4-5, (**d**) PVDF-5-5, and (**e**) PVDF-7-5. Scan size: 5 × 5 µm^2^. R_a_ represents the average of surface roughness.

**Figure 3:**
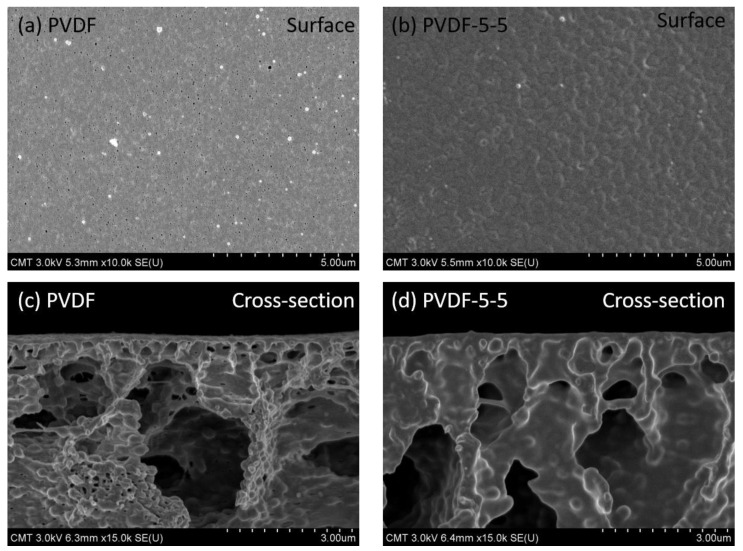
Surface and cross-section SEM images of unmodified PVDF membrane (**a**,**c**) and modified PVDF-5-5 (**b**,**d**).

The morphology of the membrane surface is generally prone to significant alteration post UV-grafting method; hence, AFM imaging was utilized to observe such surface structure transformation. The corresponding 3D AFM images and their average roughness value R_a_ are presented in Figure 2. The unmodified PVDF (a) exhibited the lowest roughness value, 10.7 ± 0.57 nm. Herein, the surface structure of unmodified PVDF was observed to be comb-like, which is similar to the early literature [38]. However, the surface structure of the modified membrane is significantly different as compared to the hill-like modified membrane. Moreover, the modified membranes exhibited a wide standard deviation of roughness developed during the UV-grafting modification. Highest roughness (17.9 ± 4.70 nm) was observed in case of PVDF-7-5, which could have been contributed by the excess amount of AA taking part in the reaction, and no significant changes in terms of roughness were observed with variation in UV exposure time. This confirms that the roughness was proportional to PAA chain density rather than the chain length during the grafting process. The morphology of AFM analysis results in combination with the ATR–FTIR results confirmed that PAA was successfully introduced onto the PVDF membrane. Furthermore, in the SEM images (Figure 3), the unmodified PVDF-5-5 membrane exhibited significant wave-like structure covering the porous surface of pristine PVDF membrane. These results were consistent with the AFM results. As evident from the cross-section images of PVDF-5-5, the PAA layer penetrated the structural pores, resulting in significant pore filling by the polymer. Thus, the PAA polymer chain not only generated the selectivity layer but also decreased the overall pore size. Consequently, the modified membrane performance may be altered due to such changes in physical structure.

**Figure 4:**
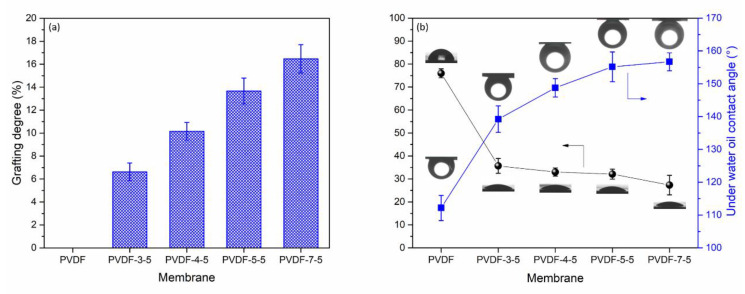
(**a**) Grafting degree, (**b**) water contact angle (black), and underwater oil contact angle (blue) of the resulting membrane.

Besides the chemical and morphology characterization, the grafting degree was used as a parameter to verify the extent of PAA loading on the PVDF membrane following various UV exposure time. The grafting degree was between 0% and 16.47%, as per the results presented in Figure 4a. The grafting degree was gradually increased, which was a direct correlation to the reaction time. Figure 4b depicts water and underwater oil contact angle for the resulting surface-modified membrane. The water contact angle can distinguish the surface hydrophilicity in correlation to the membrane performance and antifouling behavior. The unmodified PVDF had a contact angle of 75.97°, which is in good agreement with the earlier studies [27,38]. After various UV exposure time (3, 4, 5, and 7 min), the water contact angle was significantly reduced to 35.67° (PVDF-3-5), 33.00° (PVDF-4-5), 32.10° (PVDF-5-5), and 27.33° (PVDF-7-5) as compared to the unmodified membrane PVDF. Generally, several parameters, either chemical or physical properties, may affect the value of contact angles, such as surface structure, hydrogen layer, and pore size. According to the AFM results, no significant changes were observed in terms of the roughness and morphology, and hence, the reduction of the contact angle could be attributed to the hydrophilic functional group, such as carboxylic acid (–COOH) of PAA layer. The effective hydrogen layer led to the significant enhancement of the surface hydrophilicity. The underwater oil contact angle is also shown in Figure 4b with a square symbol. A hook needle was used to inject the constant volume of mineral oil from underwater. This method is able to characterize the oleophobic property of a solid surface [8,39]. The unmodified membrane exhibited the lowest oil contact angle ~110° due to the hydrophobicity of pure PVDF. The oleophobic property was gradually improved with UV exposure time. After 5 min reaction, the oil contact angle was beyond 150°. Once the oil contact angle became higher than 150°, it is described as a superoleophobic surface [39]. In prior work, Zhang et al. reported that the PAA-g-PVDF salt-induced phase inversion membrane also presented high efficiency separation permanence for oil/water and showed superoleophobic surface characteristics. Consequently, the PAA polymer chain introduced significant hydrophilic and oleophobic properties.

### 3.2. Membrane Separation Performance and Fluorescent-BSA Attachment of NF

**Figure 5:**
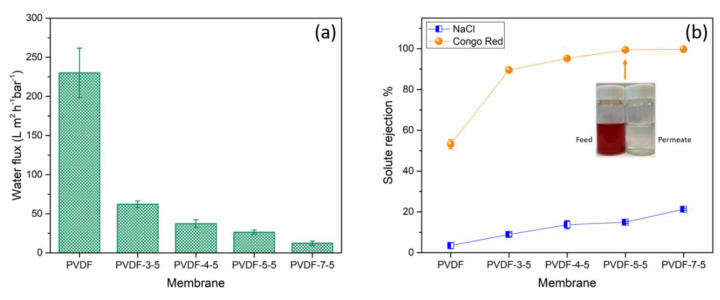
(**a**) The water flux and (**b**) Congo red and NaCl rejection of the membranes at pH 7.0. The error bar results were obtained from at least three different samples.

As per the grafting degree results (Figure 4a), a significant amount of PAA chain was deposited on the support PVDF membrane resulting in enhanced hydrophilicity on the membrane surface. The PAA chain not only created a selective layer on the surface but also reduced the pore size, as indicated in the SEM image (Figure 3), which in turn causes a rapid decrease in water flux. Herein, Figure 5a depicts the water flux of the different membrane surfaces. Pristine PVDF membrane exhibited the highest water flux of 230.14 ± 31.45 L∙m⁻^2^∙h⁻^1^∙bar⁻^1^. As the grafting degree increased, the water permeability of the modified membranes was also significantly affected by the thickness of the PAA layer and the narrow pore channel. It is evident that the PAA grafting greatly reduced the water permeability, which is consistent with our initial goal to develop loose nanofiltration membrane from the ultrafiltration membrane by using a successful UV-grafting method.

In general, NaCl is added by the textile industry as an exhausting agent or promoter of the chemical reaction [40]; consequently, the nanofiltration is a promising separation process to recycle the dye without the NaCl [20]. The dye removal and salt rejection were conducted using 100 mg/L Congo red and 1000 mg/L NaCl (Figure 5b). The rejection for Congo red of the pristine PVDF membrane was about 53.16%. When the UV-exposure time is equal to or longer than 5 min, the removal of Congo red reached up to 99.3%. The visual appearance of the feed and permeate solution are presented in Figure 5b. The high rejection of Congo red was attributed to the PAA layer and the carboxylic acid functional group, which also emitted a negatively charged surface. The NaCl rejection gradually increased but lower than 20%, which is considered beneficial for dye separation application. Because of the high Congo red rejection of PVDF-5-5, this condition will be further used to investigate the rejection against the other ion.

**Figure 6:**
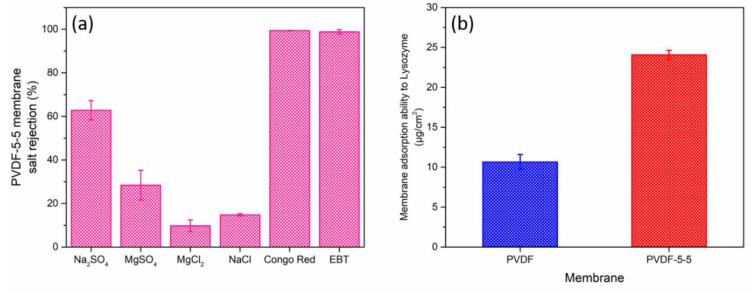
(**a**) Rejection properties of PVDF-5-5 membrane for 1000 mg/L of Na_2_SO_4_, MgSO_4_, MgCl_2_, NaCl solution, and 100 mg/L of Congo red (M_w_: 696.66) and EBT (M_w_: 461.38). (**b**) Static membrane adsorption ability to the positively charged model protein lysozyme.

Four types of salt (1000 mg/L of Na_2_SO_4_, MgSO_4_, MgCl_2_, and NaCl solution) and two negatively charged dyes (100 mg/L of Congo red and EBT) were studied, as presented in Figure 6a. Within the four salts, the modified membrane exhibited the highest rejection for Na_2_SO_4_ (~62.85%) and had the lowest rejection for MgCl_2_. The salt rejection followed this order: Na_2_SO_4_ > MgSO_4_ > NaCl > MgCl_2_. This sequence of salt rejection is common for a negatively charged NF membrane because its separation mechanism is based on Donnan exclusion [40,41]. Moreover, the Congo red and EBT rejection were above 99%, although the EBT has a smaller molecular weight, 461.38 g/mol, than Congo red, 696.99 g/mol. Its high separation efficiency was also attributed to the electrostatic repulsion between the membrane and the dye. Table 2 summarized the state-of-the-art NF membrane for the dye removal application. PVDF-PAA-5-5 (Congo red removal = 99.38%) had relatively higher water permeability than most of the membrane published in literature. It also demonstrated a higher performance than the commercially available membranes. Furthermore, the selectivity between dye and NaCl of PVDF-PAA-5-5 was superior to that of commercial membranes. These results could enhance the efficiency of the recycling or recovering process of dyes. Typically, a selective barrier made by IP reaction is denser than other modifications because of the excessively fast reaction rate between the reactants. In this study, we proposed a PAA loose structure selective layer prepared by the UV-grafting modification to construct a high dye rejection and low salt transportation channel nanofiltration membrane for the textile industry. The UV-grafting technique provides reasonable reaction speed and various controllable parameters (exposure time, intensity, and distance, and UV lamp wavelength), which can be a promising modification to precisely control and fabricate the desired membrane selectivity layer structure.

**Figure 7:**
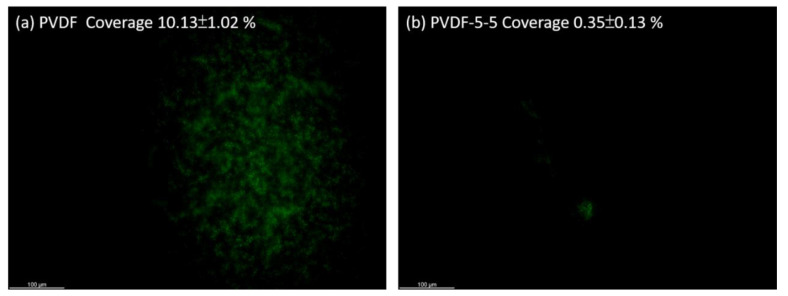
Fluorescent BSA attachment on the unmodified PVDF (**a**) and PVDF-5-5 (**b**). The membrane was exposed in the BSA solution for 3 h in PBS buffer. Scale bar: 100 µm.

The antifouling property was tested using fluorescent-BSA and lysozyme. They separately present a negative and positive charged surface in PBS buffer (pH at 7.4) due to different isoelectric points (PI) [44]. At pH ~7.4, the BSA (PI at 4.7) and lysozyme (PI at 11.) were reported with a negative and positive surface charge, respectively [32,45]. Proteins are commonly found in wastewater and other feed resources, which lead to significant membrane fouling and reduced membrane performance. BSA was used as a model protein to examine the membrane antifouling ability, which has been widely reported in early studies [32,36]. In this study, we used fluorescent-BSA, which could be easily visualized to characterize the fouling behavior (Figure 7). The green fluorescent area represents the BSA adhesion. The pristine PVDF membrane (Figure 7a) had significant foulant on the surface, and its BSA coverage was ~10.13 ± 1.02%. In contrast, the PVDF-5-5 (Figure 7b) membrane had 10 times lower coverage of BSA ~0.35 ± 0.13% than unmodified membrane. Additionally, Figure 6b shows the static membrane adsorption ability to the positively charged protein lysozyme. The PVDF-5-5 exhibits the presence of more adsorbed lysozyme as compared to the unmodified PVDF membrane, with improved hydrophilicity of PVDF-5-5 obtained from UV-grafting methods due to the presence of abundant negatively charged carboxylic acid group within the PAA layer. Hence, these indications proved that the PVDF-5-5 had a negatively charged surface, and not only had an excellent dye/salt separation performance, but also had good antifouling to the negative charge foulants and superoleophobic membrane surface.

## 4. Conclusions

In the scope of this study, an attempt was made to fabricate loose nanofiltration membrane with enhanced antifouling properties for dye removal in wastewater. Herein, PVDF membranes were surface-modified using AA as the monomer unit to grow PAA chains by following the UV-grafting method. Chemical characterization confirmed that the PAA chain was successfully grafted and covered on top of the membrane, as evident from the appearance of characteristic peaks of carboxylic acid groups and disappearance of peaks related to –CF_2_ groups. Moreover, additional morphology characterization methods confirmed the loading of PAA chains into the pores of the membranes, resulting in a significant reduction in pore diameter as well as the appearance of wave-like structure of PAA chains on the surface. Furthermore, grafting degree and contact angle measurement studies confirmed the successful introduction of the PAA chain on top of the membrane surface, along with demonstrating the hydrophilic nature and oleophobicity of the membrane due to the presence of carboxylic acid groups of PAA moiety. Dye removal efficiency of more than 99.3% and 99% was achieved using Congo red and EBT, respectively. The surface-modified nanofiltration membranes exhibited reduced water flux as compared to the base membrane, with enhanced antifouling properties, as indicated by the protein attachment experiments using fluorescent-BSA as the model protein. The resulting loose NF membrane has promising future potential to treat various wastewaters.

## Figures and Tables

**Table 1:** Elemental composition of the membrane surface from XPS analysis.

	PVDF	PVDF-3-5	PVDF-4-5	PVDF-5-5	PVDF-7-5
C1s	55.17	63.44	62.89	63.02	63.77
F1s	44.83	-	-	-	-
O1s	-	36.56	37.11	36.98	36.23

**Table 2:** Comparison of the dye separation nanofiltration membrane in the literature and commercial membrane.

Membrane	Modified Method	Water Permeability (L∙m⁻^2^∙h⁻^1^∙bar⁻^1^)	Dye Type	Dye Concentration (mg/L)	Dyes Rejection (%)	NaCl Concentration (mg/L)	NaCl Rejection (%)	Reference
PVDF-PAA ^d^	UV	26.42	CR	100	99.38	1000	14.92	This work
NF270 ^a^	IP	11.32	CR	100	99.76	1000	47.8	This work
NF90 ^a^	IP	10.49	CR	100	99.94	1000	66.49	This work
NF245 ^a^	IP	5.15	CR	100	99.81	1000	24.93	This work
DEA-PA	IP	17	CR	100	99.6	500	50.6	[7]
NFM-PIL	MMM	~15	RR	500	85	1000	7	[42]
NF-B ^c^	UV	9	SO	-	99.98	-	75	[23]
TiO_2_@PES	IP	18.1	CR	100	98	-	-	[34]
PEI-GA	Condensation ^b^	25.5	CR	100	97.1	1000	~10	[43]

CR: Congo red; RR: Reactive Red 49; SO: Safranin O. ^a^ Commercial TFC NF membrane manufactured by Dupont water solution. ^b^ Condensation reaction. ^c^ UV grafting diallyldimethylammonium chloride. ^d^ PVDF-PAA represent the PVDF-PAA-5-5 in this study.
